# Confounding-adjustment methods for the causal difference in medians

**DOI:** 10.1186/s12874-023-02100-6

**Published:** 2023-12-07

**Authors:** Daisy A. Shepherd, Benjamin R. Baer, Margarita Moreno-Betancur

**Affiliations:** 1 Clinical Epidemiology & Biostatistics Unit, Department of Paediatrics, The University of Melbourne, The Royal Children’s Hospital, Melbourne, VIC 3052 Australia; 2 Clinical Epidemiology & Biostatistics Unit, The Murdoch Children’s Research Institute, The Royal Children’s Hospital, Melbourne, VIC 3052 Australia; 3 Department of Biostatistics and Computational Biology, The University of Rochester, Rochester, New York, 14642 USA

**Keywords:** Causal inference, Skewed outcomes, Potential outcomes, Difference in medians, Confounding, Quantile regression, Inverse probability weighted, Propensity scores, G-computation

## Abstract

**Background:**

With continuous outcomes, the average causal effect is typically defined using a contrast of expected potential outcomes. However, in the presence of skewed outcome data, the expectation (population mean) may no longer be meaningful. In practice the typical approach is to continue defining the estimand this way or transform the outcome to obtain a more symmetric distribution, although neither approach may be entirely satisfactory. Alternatively the causal effect can be redefined as a contrast of median potential outcomes, yet discussion of confounding-adjustment methods to estimate the causal difference in medians is limited. In this study we described and compared confounding-adjustment methods to address this gap.

**Methods:**

The methods considered were multivariable quantile regression, an inverse probability weighted (IPW) estimator, weighted quantile regression (another form of IPW) and two little-known implementations of g-computation for this problem. Methods were evaluated within a simulation study under varying degrees of skewness in the outcome and applied to an empirical study using data from the Longitudinal Study of Australian Children.

**Results:**

Simulation results indicated the IPW estimator, weighted quantile regression and g-computation implementations minimised bias across all settings when the relevant models were correctly specified, with g-computation additionally minimising the variance. Multivariable quantile regression, which relies on a constant-effect assumption, consistently yielded biased results. Application to the empirical study illustrated the practical value of these methods.

**Conclusion:**

The presented methods provide appealing avenues for estimating the causal difference in medians.

**Supplementary Information:**

The online version contains supplementary material available at 10.1186/s12874-023-02100-6.

## Introduction

Causal inference is a central goal of health research, aiming to assess how intervening on a given exposure impacts an outcome of interest [1]. In a perfect randomized controlled trial (i.e., with no loss to follow-up) causal effects can in principle be directly estimated by comparing the average outcome in those randomised to each exposure level. However, in observational studies estimation of causal effects requires more sophisticated methods, in particular to adjust for potential confounding due to the lack of randomisation.

With continuous outcomes, the average causal effect is typically defined as a contrast of the expected (i.e., population mean) potential outcome under exposure versus under no exposure. This is usually defined in more detail by specifying the target trial we seek to emulate with observational data [2, 3]. However, epidemiological studies may suffer from skewed outcome measures, for which the expectation may no longer be interpretable as the central value of the distribution (i.e., the point in the distribution such that there is a 50% chance of a data point lying above it). Examples of skewed outcomes are abundant in health research, particularly in areas using scale scores for measurement (e.g., self-reported quality of life via the PedsQL [4], childhood behaviour via the SDQ [5]), time-to-event outcomes in the absence of censoring (e.g., survival time), or duration of events (e.g., breastfeeding duration).

When faced with this challenge in practice, there are two predominant approaches - continue to define the estimand as a contrast of expected potential outcomes, or transform the outcome to obtain a more symmetric distribution for which the expectation is interpretable as the central value. Both approaches have their advantages, although neither may be entirely satisfactory in many practical settings. Defining the estimand using expected potential outcomes may be appropriate when the expectation is of direct interest, allowing established confounding-methods to be applied [1]. However, the utility of this estimand is context-dependent, and may not be the optimal choice if the central value of the outcome distribution is of primary interest. Transformation of the outcome to be more symmetrically distributed could be an alternative solution. However, this relies on a suitable transformation existing (which may not be feasible for highly skewed distributions) and makes interpretation of the causal effects more complex than interpretation in the original scale (e.g., log-years instead of years).

An appealing alternative could be to define the causal effect using a contrast of median potential outcomes (i.e., the causal difference in medians). In fact, the causal effect has been generally defined as a contrast of any functional of the distributions of counterfactual outcomes under different exposure values [6]. However, despite being a widely acknowledged concept, there is limited availability and awareness of confounding-adjustment methods to estimate the causal difference in medians in practice. An immediate suggestion may be to use multivariable quantile regression as explored previously [7, 8], although this relies on the strict constant-effect assumption (i.e., a constant causal effect across confounder substrata) which may be too simplistic in practice. A handful of previous studies have acknowledged the need for less restrictive methods, e.g., g-methods, and presented derivations of approaches to estimate causal effects as contrasts of distribution quantiles more generally. A study by Zhang et al. (2012) derived a number of methods - a quantile regression estimator, an inverse probability weighted estimator, and a stratified estimator using propensity scores [7]. A more recent study in the field of environmental science defined a novel “overlap weighting” estimator using a class of balancing weights from functions of the propensity score model to weight each group to a selected target population [8]. These approaches are singly robust (i.e., relying on correct specification of the respective model), with a handful of doubly robust methods [7, 9–11] also proposed.

Despite their greater ease in understanding and implementation relative to doubly robust methods, application of these singly robust methods for estimating the causal difference in medians remains scarce in epidemiological research. The current discussion and evaluation of such methods is relatively limited, which has potentially led to a lack of awareness in their existence. Furthermore, to the best of our knowledge, the use of g-computation in the context of medians has not been widely discussed, let alone studied in relation to other approaches. In addition, there has been limited investigation of how these methods perform in realistic settings across various scenarios, specifically in terms of the degree of skewness in the outcome which has only been explored minimally and not for all the methods considered here [8].

In this paper we aim to describe, evaluate and compare singly robust confounding-adjustment methods to estimate the causal difference in medians, intending to increase understanding of their utility and encourage application in practice where appropriate. We focus on singly robust methods due to their wider accessibility and ease of implementation, thus aiming to bridge the gap into practice where appropriate. We revisit existing doubly robust methods for the causal difference in medians within the Discussion section of this manuscript.

We begin this paper by defining the causal effect of interest alongside an illustrative example from the Longitudinal Study of Australian Children (LSAC) [12], before outlining the confounding-adjustment methods considered. We then report findings from a simulation study motivated by the LSAC example, in addition to demonstration of the methods applied to the LSAC data. We conclude by summarising the key findings, strengths, limitations and practical recommendations.

## Defining the causal effect using medians

Consider an observational study with continuous skewed outcome variable *Y*, a binary exposure variable *A*, and a vector of *K* confounder variables $\varvec{C}$. We assume $\varvec{C}$ includes only binary or continuous variables, noting that categorical confounders can be represented as a set of binary indicators. For simplicity of discussion, we have restricted *A* to be binary and assume that no variable is subject to missingness.

The example used throughout this paper involves data from 4882 children from a nationally representative longitudinal cohort study (LSAC) [12, 13]. Children aged 4-5 years were recruited in 2004 (wave 1; approved by the Australian Institute of Family Studies Ethics Committee), with follow-ups every two years in subsequent waves. The example investigation examined the impact of maternal mental health on a child’s behaviour in early childhood in Australian families. The exposure (*A*) was a binary indicator of probable serious maternal mental illness, with the outcome (*Y*) being the child’s behavioural difficulties as measured by the Strengths and Difficulties Questionnaire [5] (SDQ). Higher SDQ scores indicate increased behavioural difficulties, with scores being positively skewed in the general population of Australia children, for which LSAC is a representative sample (see Supplementary Fig. 1 for distribution of SDQ in LSAC). Potential confounders ($\varvec{C}$) included demographic information about the child and mother (see Table 1 for a full description of all variables).

**Table 1:** Overview of variables from the Longitudinal Study of Australian Children (LSAC) example [12, 13] examining the impact of maternal mental health on a child’s behavioural difficulties in early childhood. The exposure and confounders were recorded at wave 1 (2004), with the outcome variable recorded at wave 3 (2008)

Role	Variable	Values and additional details
Outcome *Y*	Behavioural difficulties score	Range 0-40; Strengths & Difficulties Questionnaire [5]
Exposure *A*	Probable serious maternal mental illness	Yes:$A=1$/No:$A=0$; Yes defined as a K10 score < 4 [14, 15]
Confounders $\varvec{C}$	Sex of child	Male/Female
	Whether the child has siblings	Yes/No
	Child’s physical functioning score	Range 0-100; Pediatric Quality of Life Inventory [4]
	Behavioural difficulties score (baseline)	Range 0-40; Strengths & Difficulties Questionnaire [5]
	Maternal age	Recorded in years
	Maternal smoking status	Yes/No
	Maternal risky alcohol consumption	Yes/No; Yes defined as > 2 standard alcoholic drinks per day
	Maternal completion of high school	Yes/No
	Family financial hardship score	Range 0-6
	Consistent parenting score	Range 1-5

We define $Y^{a}$ to be the potential outcome when the exposure is set to level *a*. In the LSAC example, $Y^a$ represents the SDQ score for a child when their mother is set to have a probable serious mental illness ($a=1$) or not ($a=0$). Here we define the shorthand notation $m_a$ to denote the median (*med*) potential outcome under exposure level $A=a$, such that $m_a = med[Y^a]$ and $m_a \in \mathbb {R}$. Therefore, the causal difference in medians, denoted by $\delta$, is defined as the difference between the median potential outcomes under the two exposure levels:

1 $$\begin{aligned} \delta = med \left[ Y^{a=1}\right] - med \left[ Y^{a=0}\right] = m_1 - m_0. \end{aligned}$$

For the LSAC example, $\delta$ represents the difference in median SDQ scores if all children were exposed to maternal mental health problems compared to if none of them were exposed.

The causal difference in medians is identifiable from observational data under the assumptions of consistency, conditional exchangeability given $\varvec{C}$ and positivity henceforth referred to as assumptions (1-3) (see Supplementary Material S2 for further detail), as has been shown elsewhere [8, 16]. Whether these assumptions hold in practice is a matter of debate, however, for the remainder of this paper we assume these conditions do hold.

## Confounding-adjustment methods

Under the aforementioned assumptions, the causal difference in medians $\delta$ can in principle be estimated from observable data using methods that adjust for potential confounding. Here we introduce the confounding-adjustment methods investigated in our study, focusing on their implementation in practice.

### Multivariable quantile regression

Multivariable generalised linear regression is a common approach to estimate the average causal effect, adjusting for confounding through conditioning on the confounders. To estimate $\delta$, a natural adaptation is to use multivariable quantile regression (QR); a method for modelling the quantiles of the distribution of a random variable conditional on a set of covariates [17]. When applying this approach, a QR model is fitted for the outcome variable *Y* conditional on both the exposure *A* and confounder variables $\varvec{C}$, with the $\tau ^{th}$ quantile of *Y* modelled as

2 $$\begin{aligned} Q_\tau (Y|A,\varvec{C}) = \beta _0(\tau ) + \beta _1(\tau )A + \varvec{\beta }_{\varvec{3}}^{\textrm{T}}(\tau )\varvec{C}, \end{aligned}$$

By setting $\tau =0.5$, the coefficient of the exposure variable $\beta _1(0.5)$ encodes the difference in the conditional median outcome between exposure groups for every level of $\varvec{C}$ (confounder strata), i.e., $med[Y|A=1, \varvec{C}=\varvec{c}] - med[Y|A=0, \varvec{C}=\varvec{c}]$. Under assumptions (1-3), the assumption of a constant causal difference in medians across confounder strata and assuming the QR model is correctly specified, the estimated exposure coefficient $\hat{\beta }_1(0.5)$ has been shown to be a consistent estimator for the causal effect $\delta$ [8].

Quantile regression is a widely-applied and accessible method in practice, with implementation readily available in statistical software (e.g., the *quantreg* package in R [18]). However, the assumption of a constant causal effect across confounder strata may be too simplistic, and thus less-restrictive confounding-adjustment methods may be required in practice.

### IPW estimator

An alternative approach uses the framework of inverse probability weighting (IPW) to create a pseudo-population in which the distribution of $\varvec{C}$ is balanced between exposure groups, such that the association between *A* and *Y* in the pseudo-population provides an unbiased estimate for the causal effect of *A* on *Y* [19, 20]. To create the pseudo-population, observations are re-weighted in a way that is inversely proportional to the probability of the observed exposure conditional on the confounding variables via calculation of inverse probability (IP) weights. These probabilities are estimated from a model for the propensity score (PS) defined as $\pi (\varvec{c}) = P(A=1|\varvec{C}=\varvec{c})$.

A study by Zhang et al. (2012) derived an IPW estimator for the causal difference in medians and other quantiles of the potential outcome distribution [7]. Applying this approach in the context of medians, $m_a$ is estimated as the solution to the equation

3 $$\begin{aligned} \sum _{i=1}^n \hat{W}_{a,i} I(Y_i \le m_a) = 0.5, \end{aligned}$$

where $\hat{W}_{a,i}$ denotes the estimated weight for observation $i=1,\ldots ,n$ under exposure level $A_i=a$. The weights are defined as $W_{a,i}=I(A_i=a)/[nP(A_i=a|\varvec{c}_i)]$ and calculated using estimates of the propensity score, $\hat{\pi }(\varvec{c})$, obtained via a suitable regression model (e.g., a logistic regression model for *A* conditional on $\varvec{C}$). Following the suggestion of Zhang et al. (2012), normalised weights $\hat{W}^*_{a,i}$ are preferred to improve finite-sample performance [7], and are calculated by dividing each weight by the sum of all weights in the associated exposure group (see Supplementary Material S3 for mathematical formulation and further details).

To obtain the estimates $\hat{m}_1$ and $\hat{m}_0$, Equation 3 is solved for each exposure level *a*. In practice, statistical software can be used to solve this equation (e.g., via the *uniroot* function in R). Under assumptions (1-3) and assuming that the propensity score model is correctly specified, the difference between these two values consistently estimates the causal difference in medians $\delta$.

### Weighted quantile regression

An alternative implementation of IPW uses IP weights to fit a weighted quantile regression (QR) model, weighting the score equations of the regression as opposed to the observed outcomes (as is done via the IPW estimator described above). Using this approach, a univariable QR model for the $\tau ^{th}$ quantile of *Y* is specified as

4 $$\begin{aligned} Q_\tau (Y|A) = \beta _{0}^{*}(\tau ) + \beta _{1}^{*}(\tau )A, \end{aligned}$$

and fit using the IP weights estimated as outlined for the IPW estimator above. By setting $\tau =0.5$, the coefficient of the exposure variable encodes the difference in medians between each exposure level in the pseudo-population. Under assumptions (1-3) and assuming the propensity score model is correctly specified, the estimate $\hat{\beta }_{1}^{*}(0.5)$ has been shown to be a consistent estimator for the causal difference in medians $\delta$ [8].

Unlike the previous IPW estimator, which needs to be hand-coded at present, implementation of weighted QR is readily available within software packages (e.g., using the *weights* argument within the *rq* function in R [18, 21]).

### G-computation

G-computation is another popular confounding-adjustment method, arising from the g-formula which states that under assumptions (1-3), the marginal density of $Y^a$ can be identified from observable data as [22]

5 $$\begin{aligned} f_{Y^a}(y) = \sum _{\varvec{c}} f_{Y|A,\varvec{C}} (y|a,\varvec{c}) f_{\varvec{C}}(\varvec{c}), \end{aligned}$$

which is equivalent to $\mathbb {E}[f_{Y|A,\varvec{C}}(y|a,\varvec{C})]$ where the outer expectation is over $\varvec{C}$, where *f* denotes a density function. Intuitively, the right-hand side is standardising the conditional density under an exposure value to the distribution of the confounders in the whole sample, thus addressing the imbalance in confounders between exposure groups due to non-randomisation, which enables a contrast under exposure values comparable. This result can be used to identify any functional of the marginal density of $Y^a$.

When interest is in the median potential outcome $m_a$, the g-formula implies that $m_a$ can be identified as the solution to

6 $$\begin{aligned} \int _{-\infty }^{m_a} \mathbb {E}[f_{Y|A,\varvec{C}}(y|a,\varvec{C})] dy = 0.5. \end{aligned}$$

Here we note the term inside the integral is the expectation of a density, and thus the median potential outcome is not identified by a simple aggregation across the sample (i.e., the median of the conditional medians or expectations). Instead, estimation of $m_a$ using g-computation requires estimation of the conditional density within the integral. Next we describe two possible implementations of g-computation for estimation of $m_a$ - a Monte Carlo integration-based approach and an approximate approach, denoted as *g-comp (MC)* and *g-comp (approx)*, respectively.

#### G-comp (MC)

The first implementation, g-comp (MC), uses Monte Carlo simulation to perform draws from the density $f_{Y^a}(y)$, which can then be used to estimate $m_a$ [23]. Specifically, we posit a model for the conditional density of *Y* given $A=a$ and $\varvec{C}$. We then repeatedly draw from the expectation over $\varvec{C}$ of this density, i.e., corresponding to draws from $f_{Y^a}(y)$ (based on Equation 5). The median potential outcome under exposure value *a* is estimated as the median of these draws [23].

Implementing this approach requires a model for the distribution of *Y* conditional on *A* and $\varvec{C}$ (referred to as the outcome model). In the context of a skewed outcome, one possible approach is to assume that the conditional distribution of *Y* given *A* and $\varvec{C}$ follows an approximate log-normal distribution, with the mean of the underlying normal distribution dependent on *A* and $\varvec{C}$. The g-comp (MC) method would therefore be implemented as follows:

1. Fit a linear model for $\textrm{log}(Y)$ conditional on *A* and $\varvec{C}$ to the observed data.
2. Using the fitted linear model, obtain predictions of the mean outcome (on the log scale) for every observation (where $i=1,...,n$) twice:
  - Set each observation to be exposed (i.e., set $A_i=1$) to obtain predictions $\hat{\mu }^1_i = \hat{\mathbb {E}}[\textrm{log}(Y_i)|A_i=1, \varvec{C}_i=\varvec{c}_i]$, $i=1,...,n$.
  - Set each observation to be unexposed (i.e., set $A_i=0$) to obtain predictions $\hat{\mu }^0_i = \hat{\mathbb {E}}[\textrm{log}(Y_i)|A_i=0, \varvec{C}_i=\varvec{c}_i]$, $i=1,...,n$.
3. For $a=0,1$, repeatedly perform *R* draws for each observation from a log-normal distribution parametrised with the mean (on the log scale) equal to $\hat{\mu }^a_i$ and standard deviation $\hat{\sigma }$ equal to the estimated residual deviance of the model fitted in step 1.
4. For $a=0,1$, the sample median of the combined *R* samples drawn for each of the *n* observations is used as an estimate of the median potential outcome ($\hat{m}_a$) [23].

#### G-comp (approx)

In practice, the above approach may be computationally intensive, particularly for a data set with a large number of observations. Therefore, an alternative approach, g-comp (approx), approximates $\mathbb {E}[f_{Y|A,\varvec{C}}(y|a,\varvec{c})]$ by obtaining estimates of it across a grid of candidate *y* values, denoted as $y^*$, and then solves Equation 6 numerically to obtain the estimated median potential outcome. This approach again requires a model for the conditional distribution of *Y* given *A* and $\varvec{C}$ (e.g., a log-normal model as above).

When implementing this approach, steps 1 and 2 are performed as outlined for the g-comp (MC) approach. For $a=0,1$ and for each candidate $y^*$, we then estimate $\hat{f}_{Y|A,\varvec{C}}(y^*|a, \varvec{c}_i)$ for each record *i* by assuming a log-normal density with the mean (on the log scale) equal to $\hat{\mu }^a_i$ and standard deviation $\hat{\sigma }$ (as defined in step 3 above). Averaging the conditional densities over the sample yields the estimated expectation for candidate value $y^*$:

7 $$\begin{aligned} \hat{\mathbb {E}}[f_{Y|A,\varvec{C}}(y^*|a,\varvec{c})] = \frac{1}{n} \sum _{i=1}^n \hat{f}_{Y|A,\varvec{C}}(y^*|a, \varvec{c}_i). \end{aligned}$$

By repeating this process for every candidate value $y^*$, the expectation within the integral in Equation 6 is estimated across the range of *Y*. This integral is then approximated by adding up these values cumulatively, and finding the minimum value of $y^*$ for which this sum is equal to 0.5 to estimate $m_a$.

For both g-computation implementations, the difference between the estimates $\hat{m}_1$ and $\hat{m}_0$ consistently estimates the causal difference in medians under assumptions (1-3) and assuming that the outcome model is correctly specified [23].

### Standard error estimation

Previous recommendations have advised that standard errors and confidence intervals (CI) be estimated using bootstrap procedures for ease of implementation [7]. In this study, we use this approach for all confounding-adjustment methods (using the percentile bootstrap method).

## Simulation Study

A simulation study was conducted motivated by the LSAC example to evaluate and compare the performance of the five confounding-adjustment methods described in a realistic setting and under varying degrees of skewness in the outcome variable.

### Design of the simulation study

We generated 1000 datasets consisting of 1000 records for each of four skewness scenarios considered. For each scenario, dataset and record, five confounder variables $C_k$ for $k=1,\dots ,5$ (three binary and two continuous), a binary exposure *A* and a skewed continuous outcome *Y* were generated based on variables in the LSAC data set. Unless otherwise stated, the parameters of the data generating distributions were set to values estimated from the LSAC dataset (see Supplementary Table 1 for a full outline of the variables generated and their generating distribution). Values for *A* were generated from a binomial distribution with success probability defined by a logistic regression model including $\varvec{C}$ as main effects. Values for log(*Y*) were generated from a normal distribution with the mean defined by a linear regression model including *A* and $\varvec{C}$ as predictors. Different skewed distributions in *Y* were established by setting the standard deviation in the generating normal distribution for log(*Y*) to $\sigma =0.75, 1, 1.25, 1.5$ signifying increasing skewness scenarios which we denote as Scenarios 1 to 4, respectively. Values for log(*Y*) were exponentiated to obtain the outcome value *Y*, with the distribution of *Y* being positively skewed (see Supplementary Fig. 2 for the distribution of the simulated outcome variables). Here we note that $\sigma$ characterises other properties of the outcome distribution beyond the measure of skewness (e.g., the variance depends on $\sigma$). Therefore, findings across skewness scenarios are not expected to behave in a monotonic pattern.

Data was generated under two different confounding settings - weak confounding bias (approximately 10% relative bias in the unadjusted estimate relative to the true value) and strong confounding bias (approximately 20% relative bias). Modifications to confounder coefficients in the outcome-generating model (originally based on the LSAC dataset) were used to achieve this (e.g., increasing or decreasing the coefficient as required) for both confounding settings.

The true causal difference in medians $\delta$ in each scenario was computed by empirical methods [8] (see Supplementary Material S4 and Supplementary Table 2 for further details and true values used). The exposure coefficient in the outcome-generating model was modified to ensure the true value was large enough to be estimable with the given sample size with adequate power (approximately 80%) in an unadjusted analysis.

The five confounding-adjustment methods were applied to each simulated dataset to estimate $\delta$, alongside an unadjusted contrast of sample medians across exposure groups. Specific details about model specifications and implementation are provided in Supplementary Material S4, although here we note that both the propensity score model and outcome model were correctly specified (i.e., consistent with the data generation approach).

For each confounding strength and skewness scenario, metrics assessing the performance of each method were calculated using the formulae in Morris et al. (2019) [24]. For each method, we reported the bias (defined as the difference between $\hat{\delta }$ and the true value $\delta$, averaged over the 1000 samples) in absolute and relative terms (reported as a percentage); the empirical standard error (the square root of the variance of the estimates $\hat{\delta }$ across the 1000 samples); the model-based standard error (the estimated standard errors averaged over the 1000 samples); the relative error in standard errors (defined as the difference between the model-based standard errors and empirical standard error relative to the empirical standard error; reported as a percentage); and the coverage probability (estimated as the percentage of the 1000 95% confidence intervals that contained the true value $\delta$). Monte Carlo standard errors were estimated for each metric. All analysis was conducted in R 4.0.2 [21], using the *quantreg* [18] and *boot* [25, 26] packages within self-developed code (available at https://github.com/daisyshep/CI-medians.git), with the IPW estimator implemented using R code supplied in the method’s source paper [7].

### Results from the simulation study

**Fig. 1:**
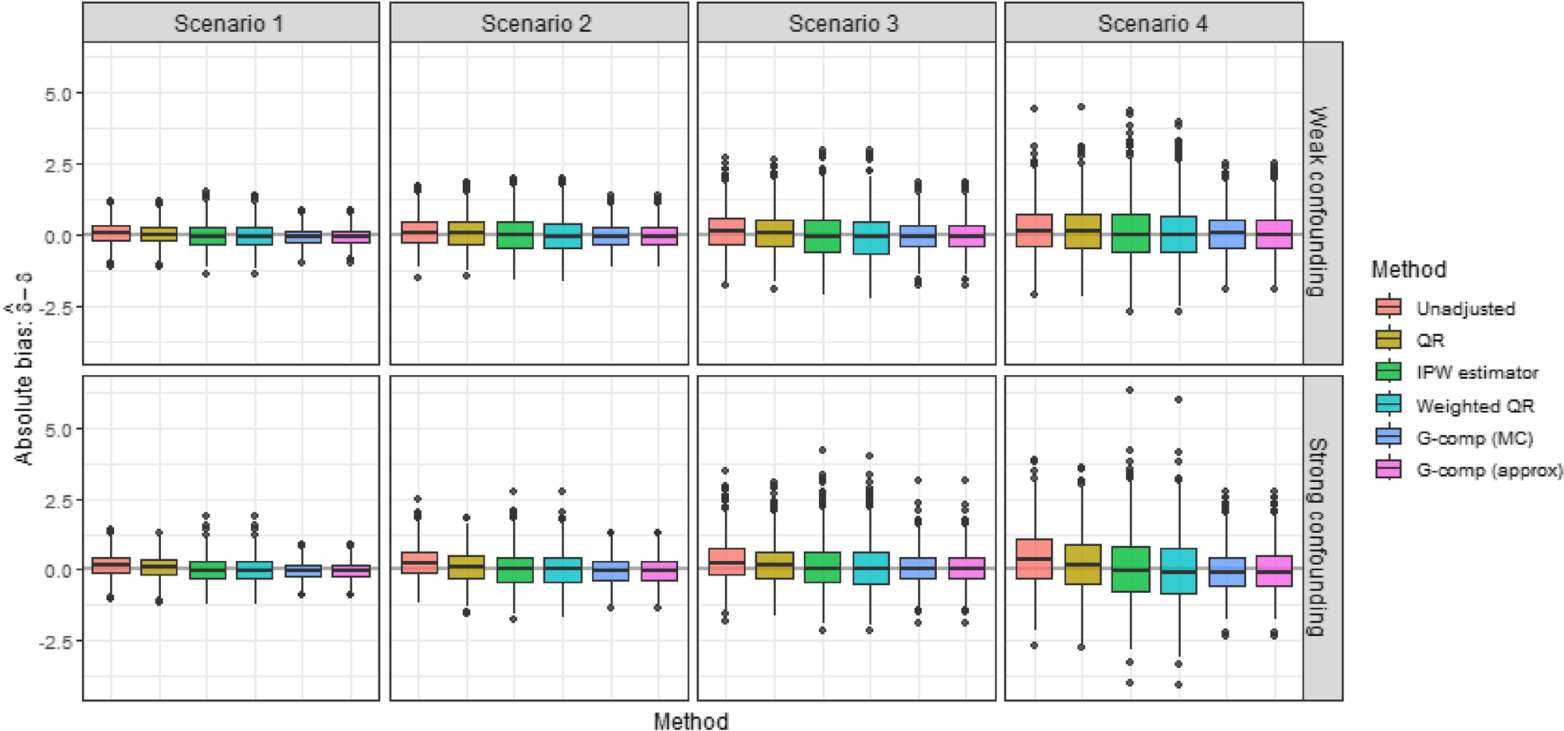
Bias in estimates of the causal difference in medians obtained under each method, for each skewness scenario and confounding setting in the simulated datasets (1000 datasets per skewness scenario). Scenarios 1 to 4 consider increasing levels of skewness, with Scenario 1 corresponding to the weakest and Scenario 4 corresponding to the highest skewness

The distribution of absolute bias in estimates is presented in Fig. 1, where it is seen that the range of absolute bias increases with increasing skewness for both confounding strengths. Estimates obtained using multivariable QR were biased across all skewness scenarios (relative bias range: 6.6% to 8.1% weak confounding, 9.0% to 14.4% strong confounding; Table 2); an expected result given the method’s strict assumption of a constant causal effect across stratum which did not hold in the data generating mechanism. In contrast, methods which relaxed this assumption (the IPW estimator, weighted QR and both implementations of g-computation) performed well across both confounding strength settings, with minimal relative bias in estimates for $\delta$ (relative bias < 5% in the majority of skewness scenarios). Both g-comp (MC) and g-comp (approx) had similar relative bias to one another across all simulation scenarios (i.e., differing by < 0.1% between implementations), with g-comp (approx) being quicker computationally to implement. Performance of the weighted approaches (weighted QR and IPW estimator) was not as similar to one another (i.e., relative bias differing by < 2.2%), although both methods yielded minimal relative bias in estimates. None of these four methods consistently outperformed the other in terms of bias, with all methods generally estimating a similar degree of relative bias within each simulation setting (combination of confounding strength and skewness scenario).

**Table 2:** Performance of confounding-adjustment methods across confounding and skewness scenarios in the simulation study with maximum Monte Carlo standard errors (SE) provided in the table footnote (see Supplementary Table 3 for all Monte Carlo SEs)

Confounding	Skewness scenario	Method	Absolute bias	Relative bias (%)	Empirical SE	Model SE	Error SE (%)	Coverage (%)
Weak	1	Unadjusted	0.090	10.07	0.369	0.381	3.21	94.60
		QR	0.059	6.63	0.384	0.375	-2.24	94.80
		IPW estimator	-0.004	-0.48	0.442	0.454	2.78	94.90
		Weighted QR	-0.023	-2.53	0.440	0.453	2.98	95.20
		G-comp (MC)	-0.026	-2.91	0.308	0.298	-3.18	93.70
		G-comp (approx)	-0.026	-2.90	0.308	0.298	-3.16	93.60
Weak	2	Unadjusted	0.123	10.08	0.526	0.528	0.32	94.90
		QR	0.099	8.13	0.540	0.517	-4.35	94.20
		IPW estimator	0.050	4.09	0.625	0.640	2.43	93.70
		Weighted QR	0.023	1.91	0.621	0.636	2.54	94.20
		G-comp (MC)	0.006	0.48	0.436	0.420	-3.60	94.00
		G-comp (approx)	0.006	0.45	0.436	0.420	-3.62	93.90
Weak	3	Unadjusted	0.160	10.00	0.684	0.727	6.27	95.60
		QR	0.114	7.14	0.693	0.695	0.31	94.70
		IPW estimator	0.019	1.17	0.806	0.870	7.94	95.00
		Weighted QR	-0.020	-1.23	0.803	0.864	7.62	95.20
		G-comp (MC)	-0.016	-1.00	0.550	0.563	2.47	95.20
		G-comp (approx)	-0.015	-0.97	0.550	0.563	2.40	95.10
Weak	4	Unadjusted	0.193	10.09	0.833	0.894	7.24	96.80
		QR	0.133	6.95	0.864	0.842	-2.62	95.50
		IPW estimator	0.123	6.45	1.032	1.098	6.40	95.30
		Weighted QR	0.081	4.22	1.017	1.091	7.24	95.50
		G-comp (MC)	0.062	3.25	0.695	0.712	2.40	95.00
		G-comp (approx)	0.063	3.28	0.694	0.710	2.20	94.90
Strong	1	Unadjusted	0.171	20.17	0.391	0.398	1.66	93.20
		QR	0.098	11.58	0.398	0.390	-2.10	94.30
		IPW estimator	0.003	0.31	0.441	0.466	5.75	95.40
		Weighted QR	-0.017	-1.95	0.440	0.464	5.33	95.30
		G-comp (MC)	-0.023	-2.65	0.308	0.306	-0.93	94.10
		G-comp (approx)	-0.023	-2.67	0.308	0.306	-0.86	94.10
Strong	2	Unadjusted	0.240	20.05	0.544	0.568	4.40	94.00
		QR	0.117	9.81	0.556	0.556	-0.03	95.00
		IPW estimator	0.030	2.55	0.621	0.673	8.34	96.00
		Weighted QR	0.004	0.33	0.617	0.670	8.61	95.40
		G-comp (MC)	-0.025	-2.08	0.446	0.441	-1.22	94.30
		G-comp (approx)	-0.025	-2.10	0.446	0.440	-1.22	94.00
Strong	3	Unadjusted	0.307	20.11	0.729	0.734	0.72	93.50
		QR	0.219	14.39	0.726	0.709	-2.32	93.70
		IPW estimator	0.117	7.68	0.847	0.897	5.89	95.40
		Weighted QR	0.082	5.35	0.843	0.891	5.77	95.50
		G-comp (MC)	0.076	4.97	0.570	0.579	1.62	95.10
		G-comp (approx)	0.075	4.93	0.569	0.579	1.75	95.20
Strong	4	Unadjusted	0.420	20.02	1.004	1.052	4.76	95.10
		QR	0.189	8.98	1.002	0.993	-0.88	94.50
		IPW estimator	0.038	1.81	1.189	1.252	5.37	95.50
		Weighted QR	-0.009	-0.43	1.180	1.241	5.19	95.50
		G-comp (MC)	-0.028	-1.34	0.759	0.806	6.11	95.40
		G-comp (approx)	-0.028	-1.32	0.760	0.791	4.12	95.70

Maximum Monte Carlo SE (performance measure): 0.038 (absolute bias), 0.018% (relative bias), 0.010 (empirical SE), 0.029 (model SE), 6.956% (relative error in model SE), 0.796% (coverage)

Both g-comp approaches yielded lower empirical standard errors comparatively to all other methods across all settings, whilst the IPW approaches (IPW estimator and weighted QR) had the largest variance across methods. Therefore, results indicated that both g-comp approaches minimised the bias and variance simultaneously.

There was little bias in estimating the standard error (SE) with the bootstrap for all methods, with a slight overestimation for the IPW and weighted QR estimator. In all settings, the coverage probability was close to nominal level for all confounding-adjustment methods (range: 93.60% to 97.70%).

## Application to LSAC

**Fig. 2:**
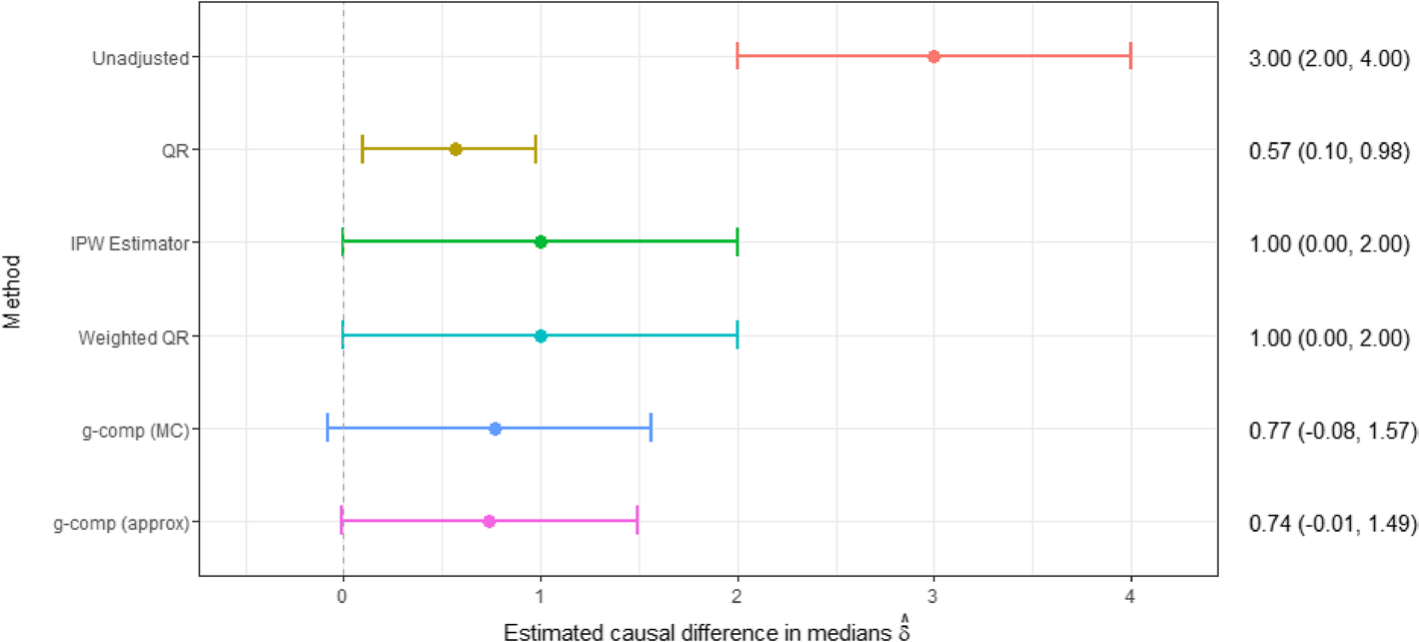
Estimates for the Longitudinal Study Of Australian Children (LSAC) example [12, 13] obtained under each confounding-adjustment method, where $\hat{\delta }$ $= \hat{m}_1 - \hat{m}_0$ is the estimated difference in median SDQ scores if all children were exposed to maternal mental health problems compared to if none of them were exposed. Point estimates and their corresponding 95% confidence intervals are presented alongside the figure

Methods were applied to the LSAC data using complete cases only ($n=3245$), with specific details about their implementation provided in Supplementary Material S5. All methods estimated the median SDQ score to be higher if children were exposed to maternal mental health problems than the median SDQ score if they were not exposed (Fig. 2), suggesting moderately increased behavioural problems as a result of maternal mental illness. Estimated effects were more consistent across the methods than observed within the simulation study, although multivariable QR yielded lower estimates of $\delta$ than the other methods. The IPW estimator and weighted QR produced equal point estimates and bootstrap CIs as did the g-computation approaches, which estimated a slightly smaller causal effect comparatively.

## Discussion

In the presence of skewed outcome data, defining the causal effect as a contrast of expected potential outcomes or transforming the outcome may not be optimal when the central value of the outcome distribution is of interest. In these cases, defining the causal effect using a contrast of median potential outcomes may be more appropriate. Despite being a widely acknowledged concept, there is scarce availability and awareness of confounding-adjustment methods to estimate this parameter in non-randomised studies. A handful of previous studies have proposed approaches to estimate causal effects defined as contrasts of distribution quantiles more generally [7, 8], but investigation of these methods and their application in health and medical studies remain scarce.

In this paper we aimed to address this gap by describing and evaluating methods identified from previous literature (multivariable quantile regression [8], an IPW estimator [7] and weighted quantile regression [8]) alongside two implementations of g-computation that, to the best of our knowledge, have not been widely described in the context of a study like LSAC or studied alongside other methods. The confounding-adjustment methods investigated were selected and described with a key focus on their accessibility and ease of implementation in a practical setting, with code made available, to encourage their use in practice where applicable.

Results from the simulation study indicated varied performance of the confounding-adjustment methods when estimating the causal difference in medians. As anticipated, the multivariable QR was too simplistic for the realistic setting reflected in our simulated datasets and produced biased estimates. The IPW estimator, weighted QR and both implementations of g-computation yielded estimates with minimal bias, with g-computation additionally minimising the variance in estimates; an expected observation as IPW estimates tend to be more variable than those obtained via g-computation [27]. We also note that the g-comp (approx) implementation was computationally more efficient than g-comp (MC) provided a suitable $y^*$ range was used.

These findings need to be interpreted in light of the fact that under our data generation approach, both the propensity score model (used for the IPW estimator and weighted QR) and outcome model (used for g-computation) were correctly specified; a critical assumption when applying the singly robust methods as noted by Zhang et al. (2012) in the case of IPW [7]. However, in the context of the medians, the outcome model for g-computation relates to specifying a model for the whole outcome density. In practice, correctly specifying this model may be harder to achieve than a correctly specified propensity score model, and thus could be considered a stronger assumption than for the weighted methods.

A strength of this work was the design of our simulation study motivated by the LSAC example, allowing us to investigate the performance of these methods in a realistic scenario. Further we investigated varying skewness scenarios alongside two different strengths of confounding bias resulting in a more complex and realistic study than those explored in previous papers [7]. Additionally, our inclusion of the g-computation approach (under two implementations) in an accessible and clear manner, has brought light to a little discussed approach.

A potential limitation of our study was the restriction to singly robust methods only, which rely on correct specification of the respective model. Previous studies have presented a handful of promising doubly robust methods, which combine both a model for the outcome and a model for the exposure and rely on only one of the models being correctly specified to obtain a consistent estimator [7, 9–11]. Doubly robust methods have not yet been evaluated in a range of complex and realistic scenarios as is done here for singly robust methods. Furthermore, their implementation is not as accessible or readily available in software as the singly robust methods presented here, and therefore their application remains scarce in practice. Both of these factors would be useful to pursue in future work. We also note that the outcome distributions explored within this study were all heavily right-skewed, so results may represent an extreme representation of the methods’ performance under heavily skewed outcome data.

Finally, we reiterate the need for the causal estimand of interest to be driven by the research question at hand. Even in the presence of skewed outcome data, it may be appropriate or preferred to define the causal effect using a contrast of expected potential outcomes. Alternatively, if a conditional average treatment effect is of relevance or interest in a particular application, the skewness of the marginal outcome distribution may not be a consideration as the conditional distribution may not be skewed anymore.

## Conclusion

When estimating the causal difference in medians, the IPW estimator, weighted QR and both implementations of g-computation present promising approaches, provided a richly specified model is used such that correct specification of the propensity score model or outcome model is likely. Implementations of the IPW estimator and weighted QR methods are readily available and accessible (e.g., *rq* function in R [21], open-source code of the IPW estimator [7]). Implementations of the g-computation approach are not as readily available, but we have provided source code which can guide practitioners in the implementation of this method (available at https://github.com/daisyshep/CI-medians.git). Overall, these methods provide appealing alternatives for estimating the causal difference in medians and avoid the stringent constant-effect assumption of multivariable quantile regression, enhancing our capability to obtain meaningful causal effect estimates with skewed outcome data.

### Supplementary Information

**Additional file 1.** This file contains the Supplementary Material. This includes: Supplementary Figure 1 (distribution of the SDQ scores in the LSAC case study); Supplementary Figure 2 (distribution of the outcome variable used within the simulation study); Supplementary Table 1 (details on the models used to generate variables for the simulation study); Supplementary Table 2 (true values for the causal difference in medians from the simulation study); Supplementary Table 3 (Monte Carlo standard errors for performance estimates from the simulation study). Additional detail to support the main manuscript is provided in Supplementary Material sections S1-S5.

## Data Availability

Data used for the LSAC illustrative example within this paper are available from the Department of Social Services (DSS) with access provided by the National Centre for Longitudinal Data (NCLD). Restrictions apply to the availability of these data, which were used under license for this study. Data are available at https://dataverse.ada.edu.au/dataset.xhtml?persistentId=doi:10.26193/F2YRL5 with the permission of the DSS. The simulated data sets are not publicly available, but the relevant R scripts can be provided upon reasonable request by contacting the corresponding author (D.S).
