# Supplementary material for: Confounding-adjustment methods for the causal difference in medians
Source: BMC Med Res Methodol. 2023 Dec 7;23:288. doi: 10.1186/s12874-023-02100-6 (PMC10702096; doi:10.1186/s12874-023-02100-6)
Supplement: Supplementary file 1 — Additional file 1. This file contains the Supplementary Material. This includes: Supplementary Figure 1 (distribution of the SDQ scores in the LSAC case study); Supplementary Figure 2 (distribution of the outcome variable used within the simulation study); Supplementary Table 1 (details on the models used to generate variables for the simulation study); Supplementary Table 2 (true values for the causal difference in medians from the simulation study); Supplementary Table 3 (Monte Carlo standard errors for performance estimates from the simulation study). Additional detail to support the main manuscript is provided in Supplementary Material sections S1-S5. [file 12874_2023_2100_MOESM1_ESM.pdf]

Confounding-adjustment methods for the causal difference in medians

Supplementary Material

Abbreviations: LSAC, The Longitudinal Study of Australian Children; SDQ, Strengths and Difficulties Questionnaire; QR, quantile regression; IP, inverse probability; IPW, inverse probability weighted; PS, propensity score

Contents

S1 The LSAC case study

S2 Defining the causal effect

S2.1 Identifiability assumptions

S3 IPW estimator

S3.1 Specification of normalised weights

S4 Additional simulation study details

S4.1 Data generation

S4.2 True causal difference in medians

S4.3 Implementation of confounding-adjustment methods in simulation study

S4.4 Monte Carlo standard errors

S5 Implementation of confounding-adjustment methods in LSAC example

References

S1 The LSAC case study

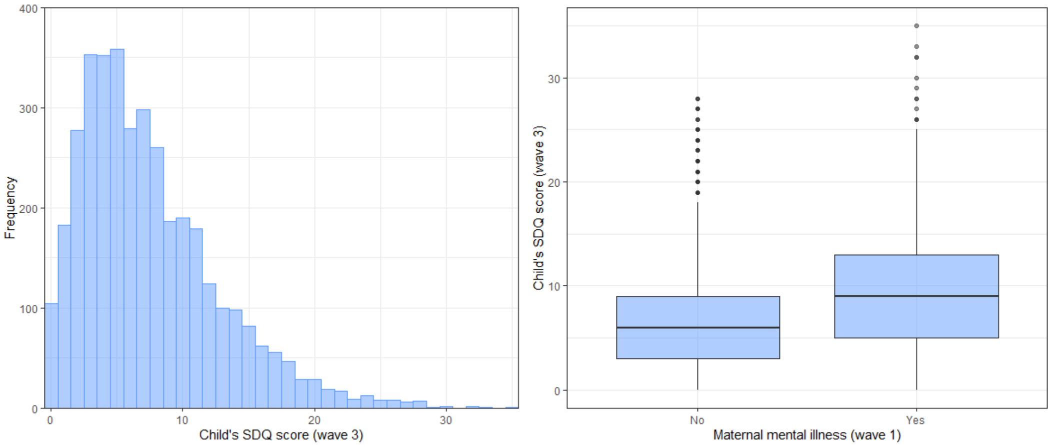

Supplementary Figure 1: Distribution of the Strengths & Difficulties questionnaire (SDQ) scores at wave 3 for the Longitudinal Study Of Australian Children (LSAC) case study.[1, 2]

S2 Defining the causal effect

To understand the formulation involved for the confounding-adjustment methods, we add further detail for the definition of the causal effect of interest (the causal difference in medians). Here we note that the median potential outcome under exposure level $A = a$ is defined as the solution to

$$\int_{-\infty}^{m_a} f_{Y^a}(y) = 0.5, \tag{S.1}$$

where $f_{Y^a}(y)$ is the density function of Y^a, or alternatively as the 50th centile of the cumulative distribution function (CDF) of Y^a, $F_{Y^a}(y) = P(Y^a \leq y)$. Under the assumption that the CDF is continuous and strictly increasing for $a \in 0, 1$, then $m_a = F_{Y^a}^{-1}(0.5)$. Therefore the causal difference in medians, denoted by δ, is defined as the difference between the median (denoted as med) potential outcomes under the two exposure levels:

$$\delta = F_{Y^{a=1}}^{-1}(0.5) - F_{Y^{a=0}}^{-1}(0.5) = med[Y^{a=1}] - med[Y^{a=0}] = m_1 - m_0. \tag{S.2}$$

S2.1 Identifiability assumptions

The causal difference in medians is identifiable from observational data under the following identifiability assumptions. Firstly we require the consistency assumption, which states that the exposure A corresponds to a well-defined intervention that in turn corresponds to the versions of the exposure in the data.[3] Under these conditions, the potential outcome Y^a is well-defined and would be equal to Y if an individual received exposure level $A = a$ (assumption 1). Secondly, we require the

conditional exchangeability assumption given the selected set of confounders, which states that the potential outcome Y^a is independent of the received exposure A given $\boldsymbol{C}$, i.e., $Y^a \perp\!\!\!\perp A|\boldsymbol{C}$ (assumption 2). Thirdly, we require the positivity assumption, which states that every individual in the population has a positive probability of being exposed or unexposed, that is $P(A = a|\boldsymbol{C} = \boldsymbol{c}) > 0$, for all $\boldsymbol{c}$ with positive probability of occurring (assumption 3).

S3 IPW estimator

Here we provide further explanation and details supporting the IPW estimator outlined in the main manuscript. The IPW estimator in Equation 3 from the main manuscript can be derived based on the following reasoning. Given assumptions (1-3) hold (as outlined in Section above), then for a given exposure level a the cumulative distribution function is equal to[4]

$$F_{Y^a}(y) = \mathbb{E}\left[\frac{I(A = a)I(Y \leq y)}{P(A = a|\boldsymbol{C})}\right]. \tag{S.3}$$

Under each exposure level $a \in \{0, 1\}$ and given estimates of the denominator probabilities from the fitted propensity score model $\pi(\boldsymbol{c})$, the expectation can be estimated using the sample average[5, 6] and can be regarded as the weighted empirical distribution of Y.[4] The IPW estimator of the median outcome value m_a is therefore defined as the solution to Equation 3 in the main manuscript. It is important to note that Equation 3 may not have a unique solution, and therefore m_a can be estimated by the value which minimises the difference between the two sides.[4]

3.1 Specification of normalised weights

In place of weights $W_{a,i}$ in Equation 3, normalised weights $W^*_{a,i}$ are advised to be use to improve finite-sample performance.[4] These normalised weights are calculated by dividing each weight by the sum of all weights in the associated exposure group, such that

$$W^*_{a,i} = \frac{I(A_i = a)}{P(A_i = a|\boldsymbol{c}_i)} \bigg/ \sum_{i=1}^{n} \frac{I(A_i = a)}{P(A_i = a|\boldsymbol{c}_i)}, \tag{S.4}$$

with the estimated values $\hat{W}^*_{a,i}$ replacing $\hat{W}_{a,i}$ in Equation 3 (in the main manuscript).

S4 Additional simulation study details

S4.1 Data generation

For each scenario, dataset and record, five confounder variables C_k for $k = 1, \ldots, 5$ (three binary and two continuous) were generated sequentially in order based on variables in the LSAC data

set. Observations for the binary confounders C_1, C_3 and C_4 (based on sex, maternal education and financial hardship, respectively) were generated from a binomial distribution with the success probability defined by a logistic regression model using all previous confounders as predictors. For the continuous confounders C_2 and C_5 (maternal age and log-transformed baseline SDQ, respectively) values were generated from normal distributions with the means defined by a linear regression model including all previous confounders as predictors. Here we note none of these models included interaction terms, as outlined in Supplementary Table 1 below.

Secondly, a binary exposure A was generated for each record from a binomial distribution with success probability defined by a logistic regression model including all confounders as main effects. A skewed continuous outcome Y was then generated for each record. Values for $\log(Y)$ were generated from a normal distribution with the mean defined by a linear regression model including the exposure and all confounders as predictors. The linear regression model was specified to include all main effects and two exposure-confounder interaction terms based on interactions observed in the LSAC example. Different skewed distributions in the outcome variable were established by setting the standard deviation in the generating normal distribution for $\log(Y)$ to $\sigma = 0.75, 1, 1.25, 1.5$ for each of the four increasing skewness scenarios, respectively. Values for $\log(Y)$ were then exponentiated to obtain the outcome value Y, with the distribution of Y being positively skewed (Supplementary Figure 2).

Supplementary Table 1: Detail on the models used to generate variables for the simulation study, with the structure based on the LSAC dataset unless otherwise specified.

Variable	LSAC variable	Generating distribution	Additional details
C_1	Sex	$C_1 \sim$ Binomial(1, 0.51)	
C_2	Maternal age	$C_2 \sim$ Normal(35.17, 5.47)	
C_3	Maternal education	$C_3 \sim$ Binomial(1, $Pr(C_3 = 1)$)	$\text{logit}[Pr(C_3 = 1)] = -1.41 + 0.78C_1 + 0.04C_2$
C_4	Financial hardship[a]	$C_4 \sim$ Binomial(1, $Pr(C_4 = 1)$)	$\text{logit}[Pr(C_4 = 1)] = -1.55 + 0.47C_1 + 0.03C_2 + 0.80C_3$
C_5	Baseline SDQ (logged)	$C_5 \sim$ Normal(μ_{C_5}, 0.63)	$\mu_{C_5} = 1.91 + 0.03C_1 + 0.01C_2 + 0.05C_3 + 0.12C_4$
A	Maternal mental health	$A \sim$ Binomial(1, $Pr(A = 1)$)	$\text{logit}[Pr(A = 1)] = -2.39 + 0.04C_1 - 0.05C_2 - 0.09C_3 + 0.51C_4 + 1.07C_5$
$\log(Y)$	SDQ score	$\log(Y) \sim$ Normal(μ_Y, σ)	$\mu_Y = 1.40 + 0.49A + 0.03C_1 - 0.01C_2 + 0.01C_3 + 0.03C_4 + 0.26C_5 + 0.12AC_1 - 0.01AC_2$ $\sigma = 0.75, 1, 1.25, 1.5$

[a]Dichotomised: No (score = 0 on original scale), Yes (score > 0 on original scale)

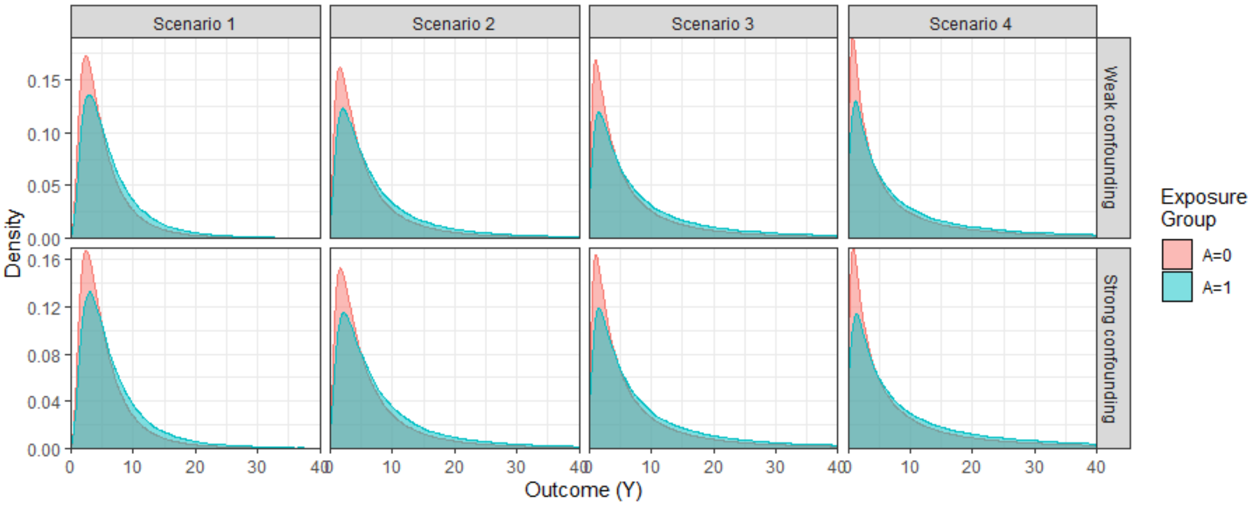

Supplementary Figure 2: Distribution of the outcome variable (Y) under each skewness scenario and confounding bias strength used within the simulation study.

S4.2 True causal difference in medians

Within the simulation study, the true causal difference in medians δ, with respect to which assess bias, was computed by empirical methods outlined in the supplementary material of Sun et al. (2021).[7] Specific details of our implementation are as follows. Initially we generated a large dataset (1,000,000 observations) per skewness scenario and strength of confounding bias (weak or strong). For each large dataset, a quantile regression model (including all main effects and exposure-confounder interactions) was fitted under different quantiles τ; we used 200 different values for τ equally distributed across the range [0.05, 0.95]. For our vector of m_a support values, we covered the range [3,8] in increments of 0.005. Different values of δ were obtained for each skewness scenario and under the two different strengths of confounding bias, as outlined below in Table 2.

Supplementary Table 2: True values for the causal difference in medians δ obtained under each skewness scenario and confounding setting.

**Confounding bias strength**	**Skewness scenario 1**	**2**	**3**	**4**
Weak	0.895	1.220	1.600	1.910
Strong	0.850	1.195	1.525	2.100

S4.3 Implementation of confounding-adjustment methods in simulation study

Here we outline the model specification and implementation of each confounding-adjustment method investigated in our study. For the multivariable quantile regression approach, the outcome model included A and $\boldsymbol{C}$ as predictors with main effect terms only, as is the default in most software. For both the IPW estimator and the weighted quantile regression approaches, the propensity score model regressed A on the confounders including main effect terms only. For the g-computation approaches, the outcome model was specified as $\log(Y)$ conditional on A and $\boldsymbol{C}$, including main effect terms and two exposure-confounder interaction terms. Here we note that both the propensity score model and the outcome model were correctly specified (i.e., consistent with the data generation approach). For g-comp (MC), we performed $R = 1000$ draws per observation. For g-comp (approx), the candidate y^* values ranged over the values [0.01, 8] in increments of 0.01.

S4.4 Monte Carlo standard errors

Supplementary Table 3: Monte Carlo standard errors of performance estimates calculated over the 1000 simulated datasets per skewness scenario and confounding bias strength under each of the confounding-adjustment methods.

Confounding	Skewness scenario	Method	Absolute Bias	Relative Bias (%)	Empirical SE	Model SE	Relative error SE (%)	Coverage (%)
Weak	1	Unadjusted	0.012	0.013	0.008	0.002	2.387	0.715
		QR	0.012	0.014	0.009	0.002	2.234	0.702
		IPW estimator	0.014	0.016	0.010	0.003	2.414	0.696
		Weighted QR	0.014	0.016	0.010	0.003	2.420	0.676
		G-comp (MC)	0.010	0.011	0.007	0.001	2.179	0.768
		G-comp (approx)	0.010	0.011	0.007	0.001	2.179	0.774
Weak	2	Unadjusted	0.017	0.014	0.008	0.004	2.607	0.715
		QR	0.017	0.014	0.009	0.003	2.364	0.702
		IPW estimator	0.020	0.016	0.010	0.007	2.768	0.696
		Weighted QR	0.020	0.016	0.010	0.007	2.750	0.676
		G-comp (MC)	0.014	0.011	0.007	0.002	2.234	0.768
		G-comp (approx)	0.014	0.011	0.007	0.002	2.234	0.774
Weak	3	Unadjusted	0.022	0.014	0.008	0.009	3.352	0.715
		QR	0.022	0.014	0.009	0.007	2.783	0.702
		IPW estimator	0.025	0.016	0.010	0.013	3.735	0.696
		Weighted QR	0.025	0.016	0.010	0.013	3.744	0.676
		G-comp (MC)	0.017	0.011	0.007	0.003	2.426	0.768
		G-comp (approx)	0.017	0.011	0.007	0.003	2.427	0.774
Weak	4	Unadjusted	0.026	0.014	0.008	0.014	4.412	0.715
		QR	0.027	0.014	0.009	0.010	3.405	0.702
		IPW estimator	0.033	0.017	0.010	0.022	5.563	0.696
		Weighted QR	0.032	0.017	0.010	0.022	5.590	0.676
		G-comp (MC)	0.022	0.012	0.007	0.006	2.947	0.768
		G-comp (approx)	0.022	0.011	0.007	0.006	2.872	0.774
Strong	1	Unadjusted	0.012	0.015	0.009	0.002	2.357	0.796
		QR	0.013	0.015	0.009	0.002	2.242	0.733
		IPW estimator	0.014	0.016	0.010	0.003	2.491	0.662
		Weighted QR	0.014	0.016	0.010	0.003	2.480	0.669
		G-comp (MC)	0.010	0.011	0.007	0.001	2.228	0.745
		G-comp (approx)	0.010	0.011	0.007	0.001	2.230	0.745
Strong	2	Unadjusted	0.017	0.014	0.009	0.005	2.582	0.796
		QR	0.018	0.015	0.009	0.004	2.397	0.733
		IPW estimator	0.020	0.016	0.010	0.007	2.879	0.662
		Weighted QR	0.020	0.016	0.010	0.007	2.869	0.669
		G-comp (MC)	0.014	0.012	0.007	0.002	2.289	0.745
		G-comp (approx)	0.014	0.012	0.007	0.002	2.291	0.745
Strong	3	Unadjusted	0.023	0.015	0.009	0.008	3.106	0.796
		QR	0.023	0.015	0.009	0.007	2.734	0.733
		IPW estimator	0.027	0.018	0.010	0.017	4.559	0.662
		Weighted QR	0.027	0.017	0.010	0.017	4.532	0.669
		G-comp (MC)	0.018	0.012	0.007	0.003	2.476	0.745
		G-comp (approx)	0.018	0.012	0.007	0.003	2.471	0.745
Strong	4	Unadjusted	0.032	0.015	0.009	0.020	5.633	0.796
		QR	0.032	0.015	0.009	0.014	4.172	0.733
		IPW estimator	0.038	0.018	0.010	0.029	6.956	0.662
		Weighted QR	0.037	0.018	0.010	0.028	6.831	0.669
		G-comp (MC)	0.024	0.011	0.007	0.008	3.304	0.745
		G-comp (approx)	0.024	0.011	0.007	0.006	2.915	0.745

S5 Implementation of confounding-adjustment methods in LSAC example

For the multivariable quantile regression approach, the outcome model included A and $\boldsymbol{C}$ (consisting of 9 confounders; Table 1 in main manuscript) as predictors with main effect terms only. The propensity score model (used for the IPW estimator and weighted quantile regression) regressed A on $\boldsymbol{C}$ including main effect terms only. For both g-comp (MC) and g-comp (approx), the outcome model was specified as $\log(Y)$ conditional on A and $\boldsymbol{C}$, including two-way interaction terms between A and three confounders (child's sex, maternal completion of high school, consistent parenting) deemed plausible based on substantive knowledge. For g-comp (MC), we performed $R = 1000$ draws per observation. For g-comp (approx), the vector of candidate y^* values ranged over the values [0.01, 18] in increments of 0.01.

References

[1] Sanson A, Nicholson J, Ungerer J, Zubrick S, Wilson K, Ainley J, et al. Introducing the Longitudinal Study of Australian Children. Australia: Australian Institute of Family Studies - Commonwealth of Australia; 2002.

[2] Christensen D, Fahey MT, Giallo R, Hancock KJ. Longitudinal trajectories of mental health in Australian children aged 4-5 to 14-15 years. PLOS ONE. 2017;12(11):1-20.

[3] Hernán MA, Robins JM. Causal Inference: What If. Boca Raton: Chapman & Hall/CRC; 2020.

[4] Zhang Z, Chen Z, Troendle JF, Zhang J. Causal inference on quantiles with an obstetric application. Biometrics. 2012;68:697-706.

[5] Horvitz DG, Thompson DJ. A generalization of sampling without replacement from a finite universe. Journal of the American Statistical Association. 1952;47(260):663-85.

[6] Robins JM, Rotnitzky A, Zhao LP. Estimation of regression coefficients when some regressors are not always observed. Journal of the American Statistical Association. 1994;89(427):846-66.

[7] Sun S, Moodie EEM, Nešlehová JJ. Causal inference for quantile treatment effects. Environmetrics. 2021;32(4):e2668.
